# A Diamond-Based Electrode for Detection of Neurochemicals in the Human Brain

**DOI:** 10.3389/fnhum.2016.00102

**Published:** 2016-03-15

**Authors:** Kevin E. Bennet, Jonathan R. Tomshine, Hoon-Ki Min, Felicia S. Manciu, Michael P. Marsh, Seungleal B. Paek, Megan L. Settell, Evan N. Nicolai, Charles D. Blaha, Abbas Z. Kouzani, Su-Youne Chang, Kendall H. Lee

**Affiliations:** ^1^Division of Engineering, Mayo ClinicRochester, MN, USA; ^2^Neurologic Surgery, Mayo ClinicRochester, MN, USA; ^3^School of Engineering, Deakin UniversityMelbourne, VIC, Australia; ^4^Physics, University of Texas at El PasoEl Paso, TX, USA

**Keywords:** diamond-based electrode, dopamine, deep brain stimulation (DBS), fast scan cyclic voltammetry (FSCV), carbon fiber microelectrode, neuromodulation

## Abstract

Deep brain stimulation (DBS), a surgical technique to treat certain neurologic and psychiatric conditions, relies on pre-determined stimulation parameters in an open-loop configuration. The major advancement in DBS devices is a closed-loop system that uses neurophysiologic feedback to dynamically adjust stimulation frequency and amplitude. Stimulation-driven neurochemical release can be measured by fast-scan cyclic voltammetry (FSCV), but existing FSCV electrodes rely on carbon fiber, which degrades quickly during use and is therefore unsuitable for chronic neurochemical recording. To address this issue, we developed durable, synthetic boron-doped diamond-based electrodes capable of measuring neurochemical release in humans. Compared to carbon fiber electrodes, they were more than two orders-of-magnitude more physically-robust and demonstrated longevity *in vitro* without deterioration. Applied for the first time in humans, diamond electrode recordings from thalamic targets in patients (*n* = 4) undergoing DBS for tremor produced signals consistent with adenosine release at a sensitivity comparable to carbon fiber electrodes. (Clinical trials # NCT01705301).

## Introduction

Deep brain stimulation (DBS) is used to treat a number of debilitating neurologic and neuropsychiatric disorders such as essential tremor, Parkinson's disease, and depression, which are linked to abnormal extracellular neurochemical concentrations (Burns et al., 1985; Sarter et al., 2007). DBS is thought to regulate or control neurochemical release (Lee et al., 2004, 2006, 2007; Bekar et al., 2008; Navailles et al., 2010; Shon et al., 2010b; Meng et al., 2011), but existing DBS devices depend on pre-programmed electrical stimulation amplitude and frequency in an open-loop configuration. To improve outcomes and reduce adverse effects, the goal of current research is an implantable closed-loop “smart” device that uses neurochemical feedback to drive stimulation parameters (Lee et al., 2009).

As a first step toward this goal, we created the Wireless Instantaneous Neurochemical Concentration Sensing (WINCS) system, a wireless, real-time, *in vivo* neurochemical monitoring system (Agnesi et al., 2009; Bledsoe et al., 2009; Shon et al., 2010a), which we have shown to be capable of monitoring neurochemical release during human DBS surgery using carbon fiber microelectrodes (CFMs) (Chang et al., 2012b). WINCS uses fast-scan cyclic voltammetry (FSCV), a well-established electroanalytical technique that can detect and measure neurochemicals *in vitro* (Rice et al., 1997; Cragg et al., 2000; Staal et al., 2004; Adams et al., 2008) and *in vivo* (Garris et al., 1999; Phillips et al., 2003; Robinson et al., 2003; Suaud-Chagny, 2004; Dommett et al., 2005; Swamy and Venton, 2007; Hashemi et al., 2009) by imposing a voltage waveform that ramps through the oxidation and reduction potentials of the species of interest while monitoring the nanoampere scale electrical current that is generated by redox reactions at specific voltages.

The integration of WINCS with a neurochemical sensing microelectrode coupled with a feedback control algorithm could serve as the foundation for a chronically implantable closed-loop DBS device. However, for such a device to be clinically useful, implanted recording electrodes must be both mechanically and electrochemically stable over the life-time of the patient. In this regard, CFMs represent a major obstacle.

Widely used with FSCV, CFMs have high sensitivity to several important neurochemicals (Cahill et al., 1996; Wightman and Robinson, 2002), fast electron-transfer kinetics (Roberts et al., 2010), relatively simple surface chemistry (McCreery et al., 1995), and low cost. At the same time, they suffer from extreme mechanical fragility, surface fouling, and a relatively small voltage range or “faradaic window” within which measurements can be made. Outside this range, water is electrolyzed into gaseous hydrogen and oxygen, preventing the detection and measurement of other compounds and rapidly destroying the electrode and surrounding tissue.

When subjected to an FSCV waveform (−0.4 V to +1.3 V vs. Ag/AgCl at 400 V/s) for an extended period of time, CFMs have been found to erode (Takmakov et al., 2010) and their mechanical integrity is eventually compromised (Keithley et al., 2011). Takmakov et al. (Takmakov et al., 2010) postulated that carbon over-oxidation causes mechanical failure via formation of carboxylic groups at the carbon surface with further oxidation through electrolysis. This leads to the generation of carbon dioxide (Takmakov et al., 2010) which dissolves in the interstitial liquid and is ultimately dispersed. Thus, extended imposition of the FSCV waveform will permanently degrade CFMs, precluding their use in chronic human implants.

Boron-doped diamond microelectrodes represent an alternative and have been developed for neurochemical detection *in vitro* (Park et al., 2005; Halpern et al., 2006; Xie et al., 2006; Patel et al., 2007; Bitziou et al., 2008; Marcelli and Patel, 2010; Singh et al., 2010; Zhao et al., 2010) and *in vivo* (Suzuki et al., 2007; Chan et al., 2009; Yoshimi et al., 2011). To-date, they have been used to: record dopamine release from the corpus striatum of the mouse brain following electrical stimulation of the medial forebrain bundle (Suzuki et al., 2007); monitor dopamine transients in response to a reward cue in the striatum of the non-human primate (Yoshimi et al., 2011); measure norepinephrine release from the sympathetic nervous system of the rat (Park et al., 2005); show important differences in brain serotonin transport function associated with genetic variability in non-human primate lymphocytes (Singh et al., 2010); and examine pharmacologically induced adenosine release from rat brain slices (Xie et al., 2006). Boron-doped diamond electrodes in these applications demonstrated excellent electrochemical properties, including a wide potential window, low baseline current, and excellent stability. However, none of these studies used FSCV, nor did they investigate the efficacy of diamond electrodes in human patients—a critical goal for their application in closed-loop DBS therapy.

To that end, we developed boron-doped diamond microelectrodes and tested their performance for real-time detection of neurochemical release using WINCS-based FSCV, both *in vitro* (to illustrate longevity) and *in vivo* (to illustrate efficacy) in human patients undergoing DBS surgery for tremor from either Parkinson's disease or essential tremor. Prior to therapeutic DBS lead placement and depending on the target of interest, an experimental diamond electrode was placed in either the ventral intermediate (VIM) nucleus of the thalamus or the subthalamic nucleus (STN), and mechanically-evoked neurochemical release was observed.

Comparing these results with the performance of CFMs, we found that while both types of electrodes demonstrate good sensitivity in detecting neurochemicals *in vitro* and *in vivo*, boron-doped diamond electrodes have increased longevity and mechanical strength, critical characteristics for reliable and safe use *in vivo*. Our results suggest that boron-doped diamond electrodes may be the solution for chronic implantation which could support basic research investigating long-term neurochemical responses in animals, and in humans, it could potentially “close the loop” in DBS.

## Results

These studies were approved by Mayo Clinic IRB (IRB #09-007441) and registered with ClinicalTrials.gov (Clinical trials # NCT01705301).

### Diamond electrode characterization

The electrodes used in this study were prepared by depositing films of polycrystalline boron-doped diamond on conically-sharpened tungsten rods using chemical vapor deposition (CVD). Electrodes were prepared in batches of 8 (as this is what the CVD reactor is designed to accommodate), and up to one batch per day was produced. Thus far, our lab has produced and characterized in excess of 500 diamond-coated electrodes. The resulting boron-doped diamond electrode tips were characterized by scanning electron microscope (SEM) (all batches) and Raman spectroscopy (select batches).

**Figure 1:**
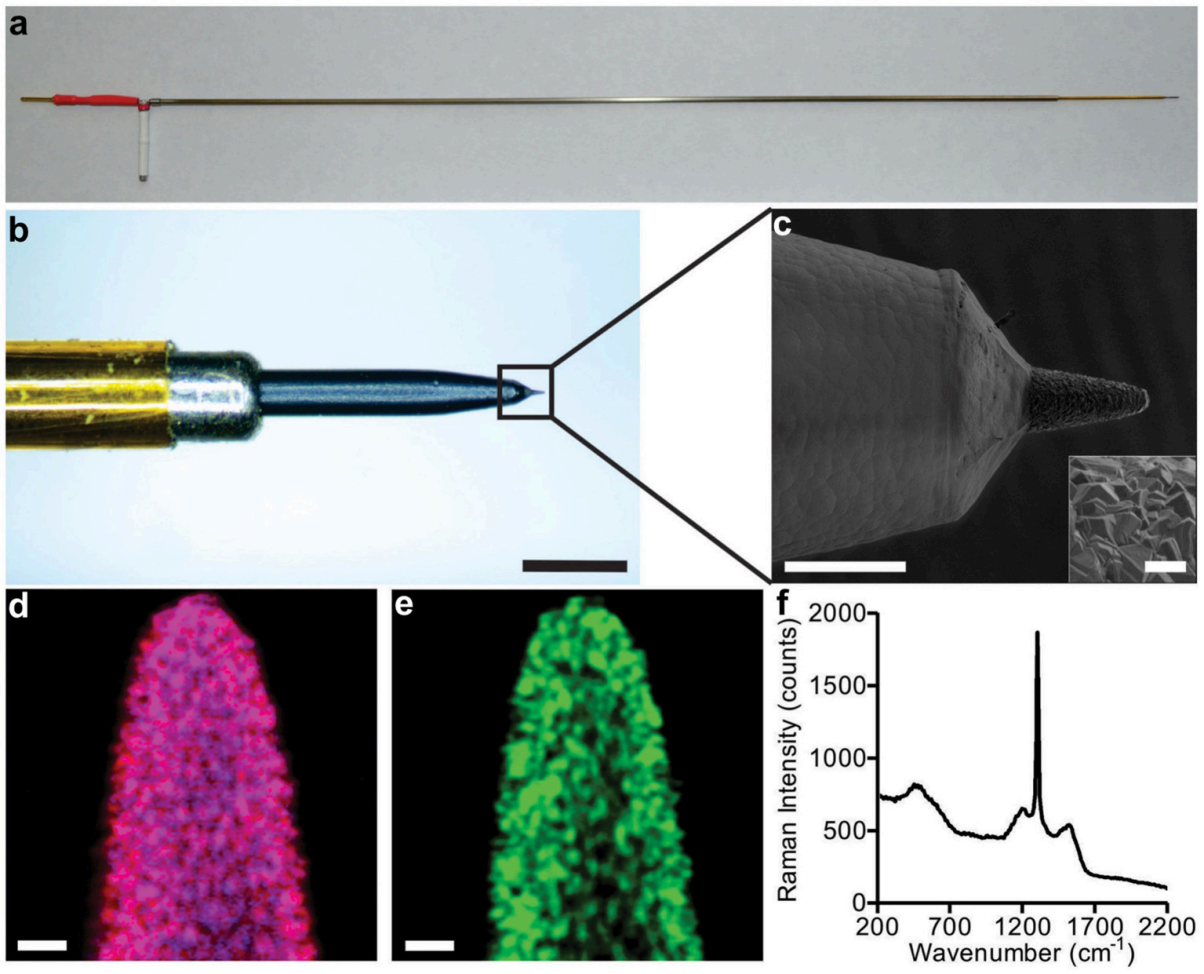
**Diamond FSCV probe construction**. Panel **(A)** depicts the overall full length electrode suitable for acute large animal and human recordings. The scale bar is 25 mm. Panel **(B)** shows a detailed optical magnification of the tip and its individual components, scale bar is 500 μm. Panel **(C)** is an SEM image, showing the transition from the parylene insulation to the diamond sensing tip, scale bar is 100 μm (crystal detail scale bar is 10 μm). Panels **(D,E)** show Raman spectral maps of the probe depicted in panel **(C)**, scale bar is 10 μm. The three color channels assigned to material constituents are: blue for boron doped diamond, red for diamond, and green for carbon sp^2^ impurities. Magenta color observed in image **(D)** is a combination of blue and red. Panel **(F)** shows the integrated Raman spectrum of Raman images in panels **(D,E)**. The Raman measurements were acquired with a 532 nm excitation source.

Imagery depicting an example electrode is illustrated in Figure 1. The boron-doped diamond films were polycrystalline, with average crystal dimension within the range of 0.5–2 μm and a film thickness of approximately 5–10 μm. Both (1 1 1) and (1 1 0) orientations were observed. The diameter of the conical electrode tips was approximately 50 μm, and the exposed length around 100 μm for a total (geometric) surface area of approximately 10,000 μm^2^. Figure 1 provides more details of the electrode tip and parylene insulation layer/diamond film interface.

To allow visual determination of material constituents, Raman microscopy was performed at the electrode tip. The results are presented in Figures 1D,E. The dominant magenta color resulting from a combination of blue (assigned to boron) and red (assigned to diamond) that is observed in Figure 1D, demonstrates a relatively uniform incorporation of boron into diamond. As diamond is composed of carbon atoms exclusively bonded to each other by sp^3^-hybridized orbitals, its characteristic Raman spectrum is a single peak at 1332 cm^−1^. Besides the existence of this sharp peak, the integrated Raman spectrum of Figures 1D,E, presented in Figure 1F, reveals other less intense bands. The broader band around 1500 cm^1^ is attributed to existence of a small amount of carbon sp^2^. The weak features around 1230 cm^−1^ and 550 cm^−1^ correlate with boron incorporation and accumulation in the diamond lattice, respectively (Bernard et al., 2004). These data confirm that the diamond-like crystal morphology observed in the SEM imagery does, indeed, correspond to boron-doped polycrystalline diamond.

### Electrode sensitivity and longevity

**Figure 2:**
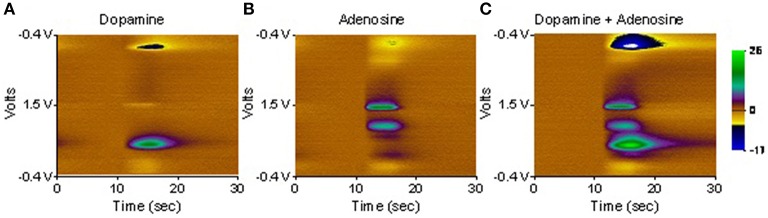
**Diamond FSCV probe exposed to dopamine at 2.5 μM (A), adenosine at 20 μM (B), and dopamine + adenosine at the same concentrations (C)**. Note that the signals of the two neurotransmitters occur at different voltages and are effectively additive. That is, the voltammogram of dopamine + adenosine is a linear combination of the voltammograms of the two individual neurotransmitters.

First, any prospective FSCV electrode must produce a voltammogram with distinct signatures that correspond to analytes of interest (specificity). For instance, both dopamine and adenosine may be encountered in the thalamus—a common DBS target—and the electrode must be able to separate their signatures. Toward this end, dopamine, adenosine, and a combination of the two were presented to a diamond electrode in a flow cell. As illustrated in Figure 2, their oxidation and reduction signatures are quite distinct, and a combination of the two analytes produces a voltammogram that is a simple linear addition of the individual voltammograms.

While the *in vivo* data presented in this work are acute (no more than 30 min per patient were allowed by the IRB-approved experimental protocol), the motivation for moving from carbon fiber to diamond is the promise of vastly-improved longevity sufficient for chronic implantation. Two metrics are of crucial importance in the construction of any electrode destined for chronic *in vivo* implantation: sensitivity of the electrode to the analyte(s) of interest, and the ability of the electrode to maintain that sensitivity after extended continuous use.

**Figure 3:**
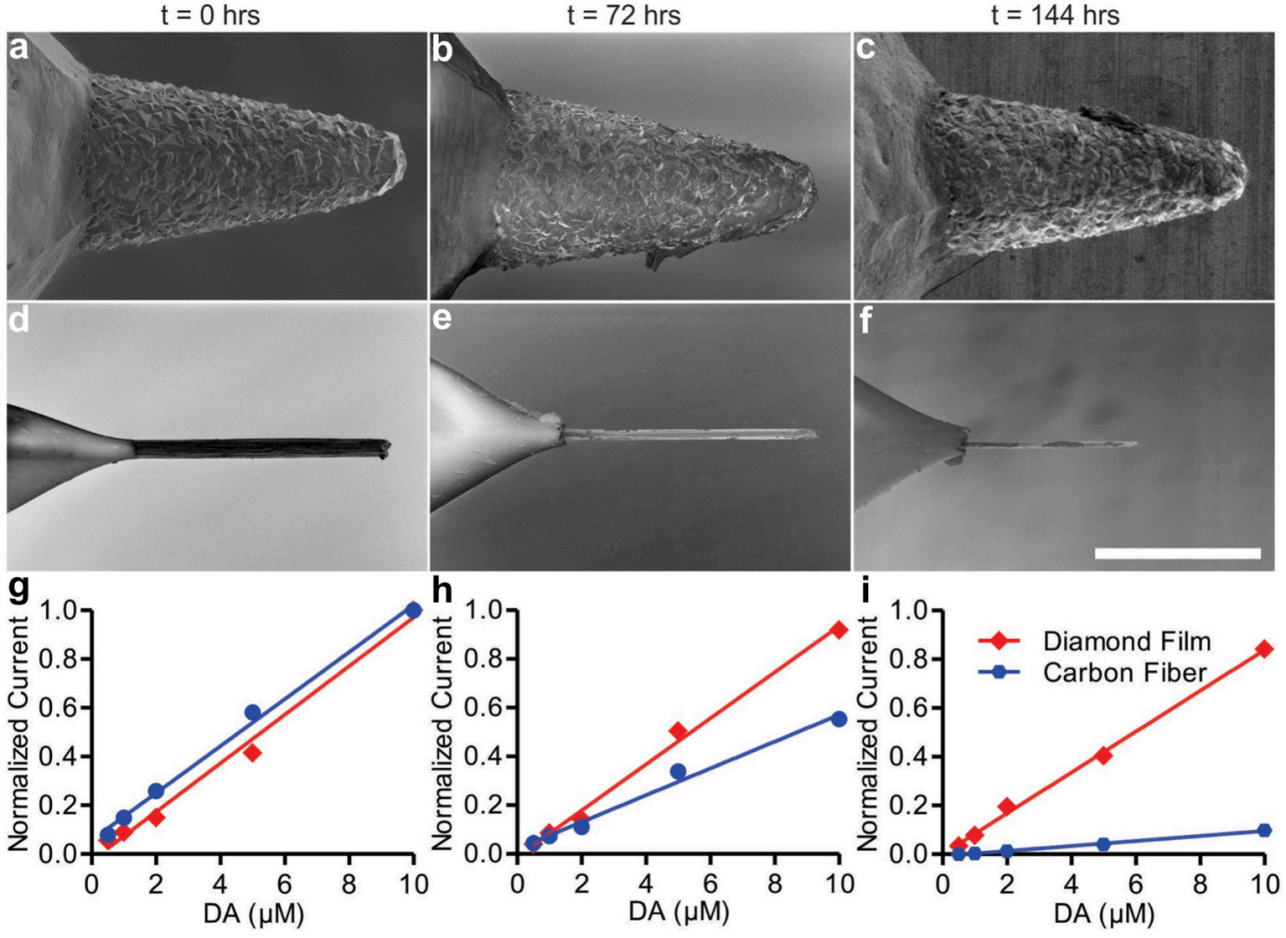
**SEM images of diamond electrode (A–C) and carbon fiber electrode (D–F)**. Panels **(A,D)** are at *t* = 0, panels **(B,E)** *t* = 72 h, and panels **(C,F)** are at *t* = 144 h. Panels **(G–I)** show diamond and carbon fiber calibration curves for the detection of dopamine at *t* = 0 h **(G)**, *t* = 72 h **(H)**, and *t* = 144 h **(I)** of continuous use.

To test these metrics, dopamine was selected as the test analyte for these *in vitro* studies, as the dopamine oxidation and reduction responses at CFMs using FSCV have been well characterized (Robinson et al., 2003). Newly-fabricated diamond electrodes were imaged with a SEM and calibrated by subjecting them to dopamine in flowing Tris buffer while applying the FSCV voltage waveform described in the Methods section. After calibration with dopamine, the electrodes were transferred to a bath of pure Tris buffer where the FSCV waveform was applied continuously for an extended period of time. CFMs (a current standard for FSCV use) were subjected to identical treatment as a control. After 72 and 144 h of continuous use at these conditions, both types of electrodes were removed from the buffer and recalibrated. After the conclusion of the experiment, scanning electron micrographs were obtained. The results of the corresponding calibrations are depicted in Figure 3.

Throughout the course of the experiment, the sensitivity decreased for both the diamond and the CFMs. The diamond film electrode average dopamine sensitivity (nA/μM) decreased 6.7% from *t* = 0 to *t* = 72 h (Figure 3H; 95% confidence interval [CI]: –3.6 to 15.8%), and 16.1% from *t* = 0 h to *t* = 144 h (Figure 3I; 95% CI: 7.8 to 23.4%). In contrast with the stability demonstrated by the diamond electrode, the CFM's calibration curve revealed marked degradation in sensitivity after 24 h. The CFM average dopamine sensitivity decreased 43.4% from *t* = 0 to *t* = 72 h (Figure 3H; 95% CI: 37.5 to 48.9%) and 89.4% from *t* = 0 h to *t* = 144 h (Figure 3I; 95% CI: 88.6 to 90.2%). After 144 h of use—5.2 million measurement cycles—the CFM had become, in effect, completely insensitive to dopamine and the calibration experiment was discontinued, while the diamond electrode retained 83.9% of its initial sensitivity.

After 3 and 6 (total) days of continuous use, both types of electrodes were subjected to imaging by SEM. The resulting SEM imagery is presented in Figures 3A–F. While the diamond electrode showed no discernible changes, the CFM had been almost completely eroded. Given the chemical simplicity of the buffer solution, this erosion is most likely due to physical dissolution of the electrode tip by electrolysis reactions at the electrode surface:

$$\begin{array}{l} \text{C}+2{\text{H}}_{2}\text{O}\to {\text{CO}}_{2}+4{\text{H}}^{+}+4{\text{e}}^{-}\text{and}∕\text{or}\\ \text{C}+{\text{H}}_{2}\text{O}\to \text{CO}+2{\text{H}}^{+}+2{\text{e}}^{-} \end{array}$$

during the period of the FSCV waveform where the CFM is acting as the anode (positive polarity) with a voltage in excess of 1.2–1.4 volts. While diamond is also an allotrope of carbon, the higher degree of covalent bonding likely leads to the above reactions occurring at much slower rates (though degradation is still thermodynamically favorable).

### Physical robustness

Another critical property of any electrode intended for human surgical use is its physical robustness with respect to mechanical damage. While electrochemical durability in a flow cell is a necessary first step to a practical electrode design, the final electrode must resist forces stronger than those exerted by tissue resistance during implantation. The tissue of the living brain, while one of the softest tissues in the body, is still firm enough to break a single carbon fiber.

To quantify their relative resilience, both electrode designs were slowly forced into a stainless steel plate while the displacement distance and exerted force were simultaneously recorded. Both electrodes deformed by approximately 100–120 μm before failing; however the failure modes were very different. The CFM exerted a fairly constant force of about 0.1–0.15 g against the plate as the carbon filament at the tip slowly bowed outwards. When the carbon fiber could bend no further, it snapped, and the force against the plate was reduced to zero.

The diamond-coated tungsten electrode tip, by comparison, did not break at any point. Rather, it slowly deformed as the sharp tip bent to one side and curled back on itself. Even after exerting 30–40 g of force (approximately 200 times more force than caused the CFM to fail), the diamond electrode remained largely intact. Both electrodes were examined by SEM following the test, and the only apparent damage to the diamond electrode was some loss of the diamond coating where the bending of the tungsten substrate occurred. The carbon fiber electrode, by contrast, was destroyed.

### Diamond electrode sensitivity *in vivo*

The results obtained regarding electrode longevity and strength strongly suggest that diamond electrodes would be superior for chronic implantation as required for FSCV sensing in an implantable closed-loop DBS system. Following these *in vitro* trials, a series of animal trials were conducted in both small (*Rattus norvegicus*) and large (*Sus scrofa*) animals to determine *in vivo* efficacy. However, to demonstrate the efficacy of diamond electrodes for their intended application, volunteer test subjects were recruited from among patients undergoing DBS lead placement surgery for tremor disorders (Parkinson's disease and essential tremor).

**Figure 4:**
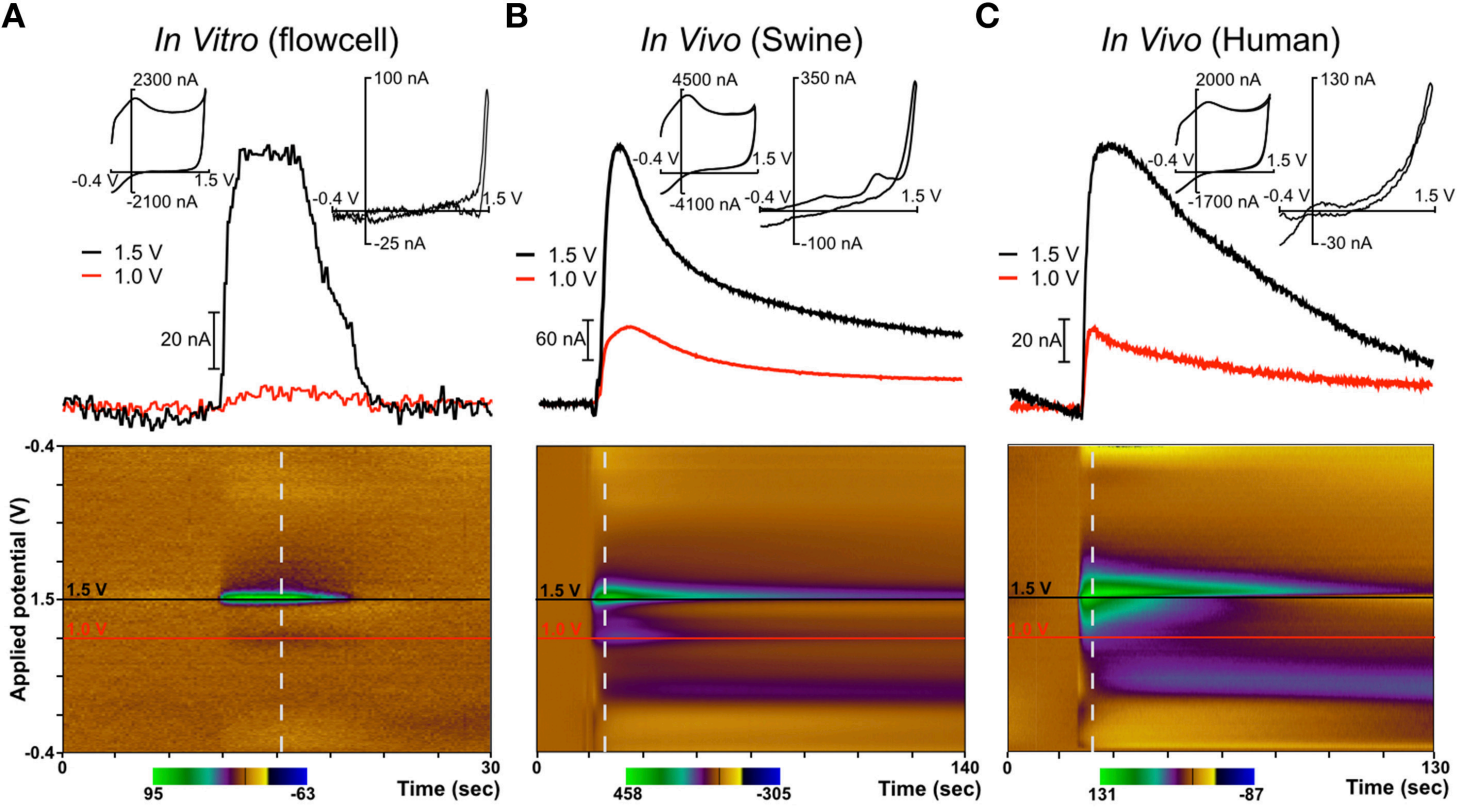
**Diamond electrode performance ***in vitro*** and ***in vivo*****. Panel **(A)** depicts a diamond electrode's response to identical *in vitro* conditions. Panel **(B)** depicts the same diamond electrode in the ventral lateral thalamus of an anesthetized pig, where an adenosine-like signature secondary to mechanical stimulation was observed. Panel **(C)** depicts the same electrode in the subthalamic nucleus of an awake human undergoing DBS lead-placement surgery for essential tremor (prior to the DBS lead placement). In panels **(B,C)**, the signature is consistent with adenosine based on the oxidation peaks near the switching potential at 1.5 V. All “diamond” data was recorded with the same specific electrode (although not in the order shown—the human data were always gathered first).

In order to minimize risk to the patients, only one parallel surgical trajectory was permitted by the IRB-approved protocol, chosen solely based on the needs of the patients' intended DBS therapy. In patients selected for these studies, the therapeutic DBS lead—a standard 4-contact lead manufactured by Medtronic (model 3387 or 3389)—was targeted at the VIM nucleus of the thalamus (*n* = 3) or the STN (*n* = 1). The diamond electrode was inserted into the target region first, prior to the therapeutic DBS electrode, and mechanically-stimulated release of the neurochemical adenosine due to the “microthalamotomy” effect (Chang et al., 2012b) was observed, as depicted in Figure 4. Although dopaminergic neurons have been shown to innervate parts of the non-human primate and human thalamus (principally dorsal aspects), only adenosine release was observed. This was likely due to the specific region of the thalamus targeted for DBS which has been shown to contain a relatively small number of dopaminergic terminals (Sanchez-Gonzalez et al., 2005) (targeting information is provided in the Supplementary Materials).

**Figure 5:**
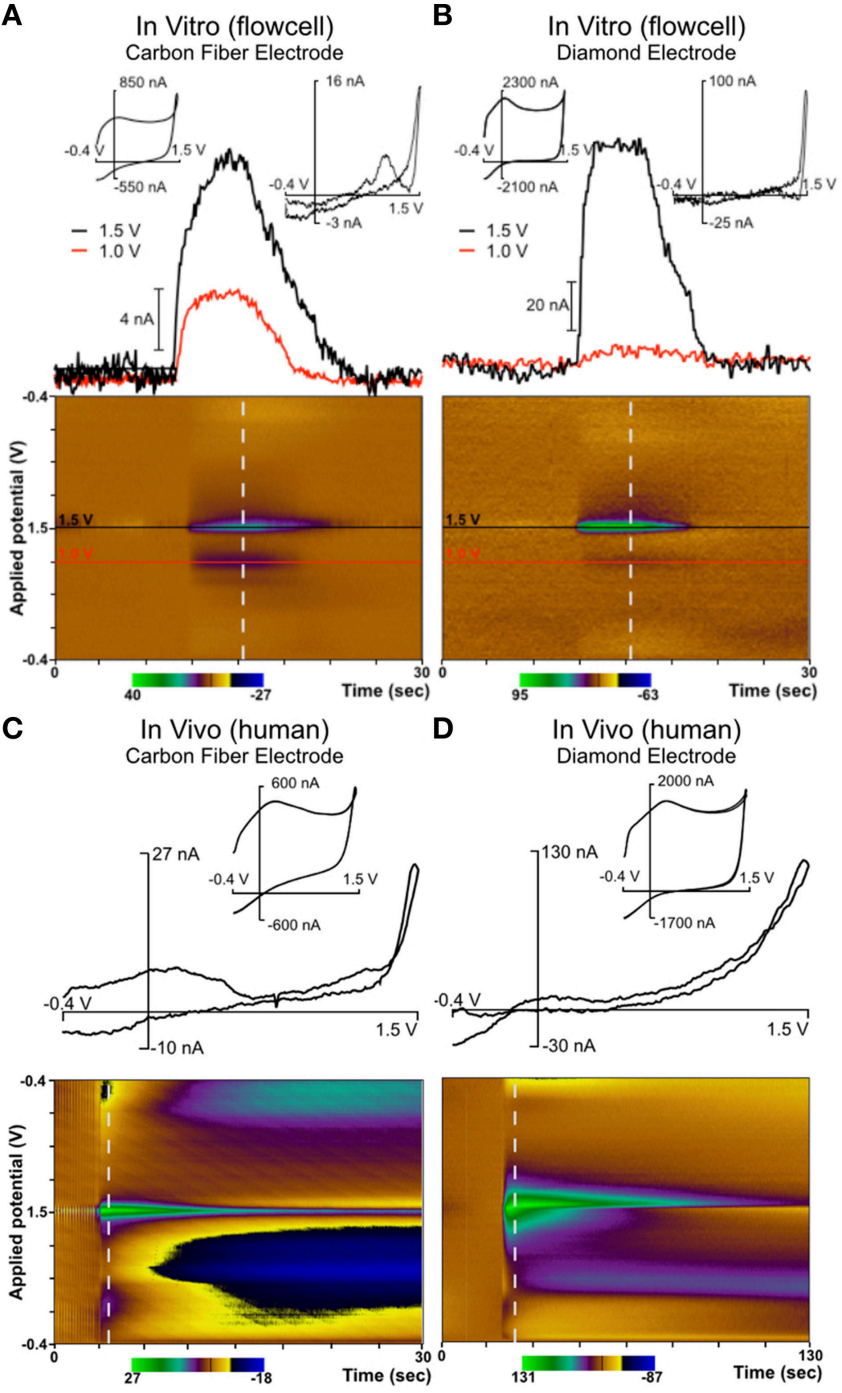
**Comparison of diamond electrode (the same electrode depicted in Figure 4) with a carbon fiber electrode in a flow cell and ***in vivo*** in the ventral intermediate (VIM) nucleus of the thalamus in a human patient**. In both *in vivo* cases, neurotransmitter release was evoked by mechanical stimulation (“microthalamotomy” effect) and showed an adenosine-like signal. **(A)** Carbon fiber electrode *in vitro*; **(B)** Diamond electrode *in vitro*; **(C)** Carbon fiber electrode *in vivo* (human); **(D)** Diamond electrode *in vivo* (human).

In these experiments in humans (and in swine) an adenosine-like signature secondary to mechanical stimulation was observed, with oxidation peaks around 1.5V and 1.0V. The additional signature around 0.5V is most likely due to a local change in pH—a known effect in FSCV recordings using CFMs (Runnels et al., 1999). This represents the first application of FSCV via diamond-based electrodes in human subjects and verifies the diamond electrode's ability to sense evoked changes in the extracellular levels of neurochemicals in the human brain. Previously, our laboratory also conducted a very similar series of experiments using CFMs in human subjects (Chang et al., 2012b). These experiments, conducted using the same protocol as the diamond experiments, also detected adenosine-like signatures secondary to mechanical stimulation, as depicted in Figure 5.

Figure 5C depicts mechanical stimulation of an adenosine-like species in the VIM of an awake human patient using a traditional CFM. This patient was undergoing DBS lead placement surgery for Parkinson's disease. Figure 5D depicts an identical experiment (same target, same protocol) conducted in a different patient using a diamond electrode. The biochemical milieu of the human brain is far more complex than the pure adenosine present in a flow cell. However, the signature oxidation peak at the potential of +1.5V is highly consistent with adenosine oxidation and was present in all cases. In the various panels of Figure 5, it is clear that the diamond electrode is less sensitive to the second oxidation peak of adenosine (around 1.0 V) than carbon fiber. The diamond electrodes are also less sensitive to the broad feature associated with pH change located around 0.5 V. However, given that the relative strength of the various oxidation peaks for adenosine oxidation products is variable even between different grades of carbon fiber (Swamy and Venton, 2007), the fact that there is some difference in behavior between carbon fiber and diamond—two entirely different carbon allotropes—is not surprising.

**Figure 6:**
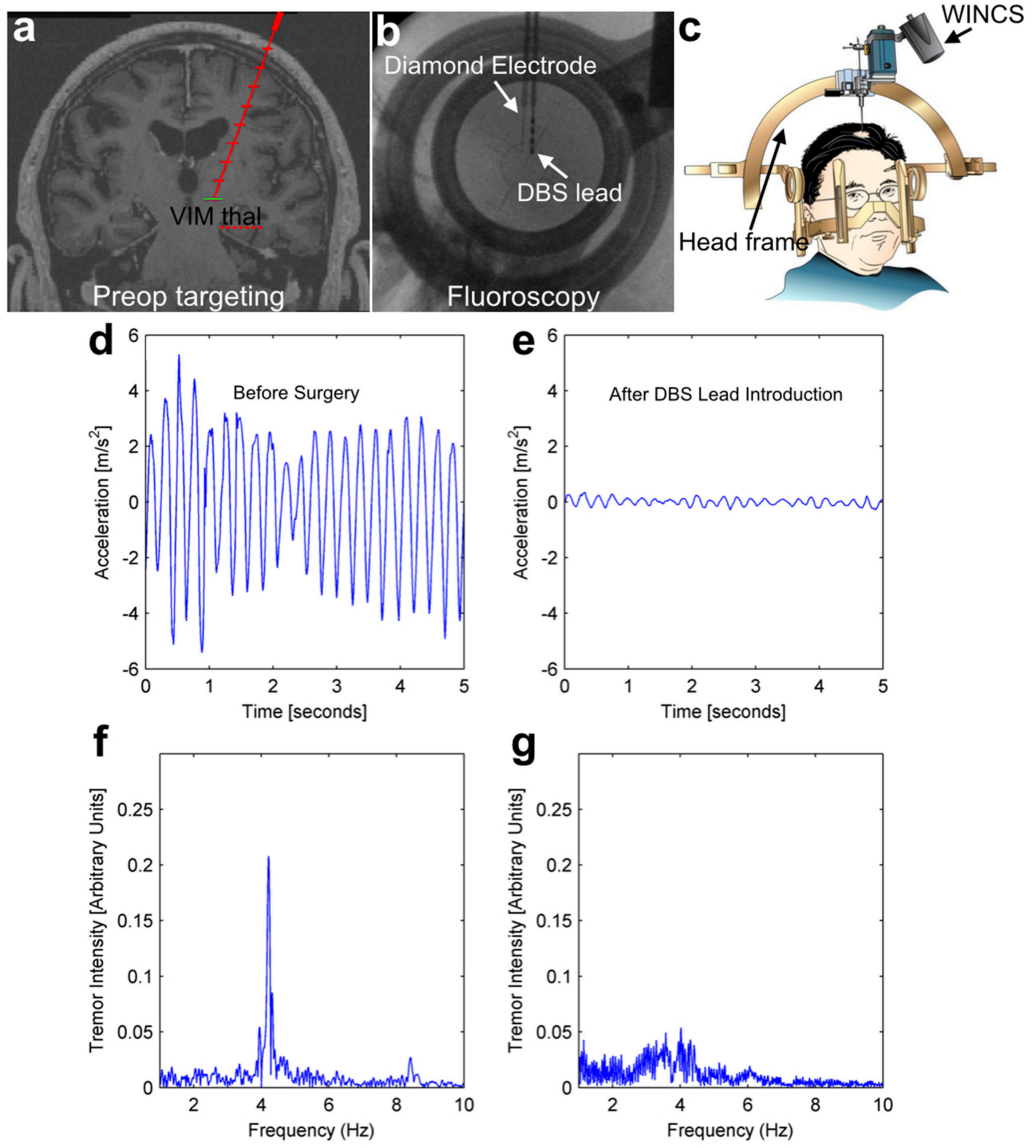
**Intraoperative tremor recordings before and after DBS lead placement**. This patient underwent DBS lead placement for essential tremor, with the diamond-based electrode placed in the ventral intermediate (VIM) nucleus of the thalamus prior to therapeutic DBS lead placement (top panels **A–C**). Throughout the surgery, the patient wore a hand-mounted wireless accelerometer to measure tremor. Prior to surgery, the patient displayed a pronounced tremor at 4.2 Hz (left panels, **D,F**), while after lead placement the mechanical stimulation alone (“microthalamotomy” effect) caused a cessation of this tremor with no applied voltage (right panels, **E,G**).

Human test subjects are ideal in the sense that they are the ultimate intended environment for a chronic sensing electrode for a closed-loop DBS system, however human trials do present some unique challenges. These challenges include the inability to pharmacologically-manipulate neurotransmitter levels, the short duration of recording that is possible, and a surgical trajectory that is defined and limited by the needs of the therapeutic procedure the patient is undergoing. All study patients were equipped with a hand-mounted wireless accelerometer. In the case of a representative patient depicted in Figure 6 (essential tremor, lead placement in the VIM), the mechanically-evoked release of the neurochemical adenosine (“microthalamotomy effect”) caused by lead placement was sufficient to produce an almost complete cessation in the patient's tremor. Prior to lead placement, this patient exhibited a strong tremor at 4.2 Hz as shown in Figures 6D,F, while this tremor is absent post-placement in Figures 6E,G.

This effect is common in human patients, even without an electrical current being applied to a stimulating electrode. The mechanical effect of lead placement alone is sufficient to ameliorate tremor. As depicted in Figures 4, 5, mechanical advancement of the electrode is also associated with adenosine-like signals detectable by FSCV. While this link is not sufficient to draw conclusions vis-à-vis mechanism, it does demonstrate that the adenosine-like signals accessible to a diamond electrode are related to the desired clinical outcome of tremor-reduction—a necessary condition for closed-loop DBS.

## Discussion

It has been demonstrated that a CVD diamond reactor has the ability to deposit films of high quality boron-doped polycrystalline diamond on tungsten electrode substrates. These diamond-coated tungsten needles can be employed to create durable and sensitive electrodes for FSCV.

After fabrication, these diamond-coated tungsten needles form complete electrodes useful for electrochemical detection of neurochemicals. Furthermore, these diamond electrodes, in conjunction with WINCS-based systems (Agnesi et al., 2009; Bledsoe et al., 2009; Shon et al., 2010a), are capable of detecting changes in extracellular concentrations of neurochemicals. Their ability to sense neurochemicals was verified with *in vivo* detection of adenosine-like signals in either the VIM nucleus of the thalamus or the STN of human patients undergoing DBS lead-placement surgery, comporting well with the findings of Chang et al. (2012b).

The FSCV sensing lifetime of diamond-based electrodes is dramatically superior to the lifetime of the current standard CFMs, showing little degradation over 5.2 million cycles (144 h at 10 Hz), while a CFM was eroded and rendered almost completely insensitive under the same conditions. If this was a chronic implant, 5.2 million scans would be roughly equivalent to 1 scan every 2 min for 20 years. While additional degradation or desensitization mechanisms could affect both electrodes *in vivo*, the electrochemical degradation seen here would certainly render carbon fiber unusable over this timespan.

In addition, the diamond electrode design exhibited a greater than 2 order-of-magnitude improvement in physical robustness with respect to mechanical damage of the tip. This robustness is critical for eventual chronic human application. Given the softness of brain tissue and the small diameter of the carbon fiber (on the order of 10 μm), even the 0.1–0.15 g of insertion force that the carbon fiber electrodes withstood will often be sufficient for acute experiments. Still, reliable use of such delicate electrodes demands precise assembly and tolerates almost no asymmetry in the application of force (i.e., insertion must be straight into the brain with no lateral forces). The stronger diamond-based electrodes are far more forgiving.

For either basic science studies of long-term neurochemical release in the brain, or the eventual construction of a closed-loop DBS device, a long-lived sensing electrode is required. Full realization of a practical closed-loop DBS system will still require additional work. First, these sensing tips must be incorporated into a flexible and implantable lead that mates with an appropriate electronics package, not unlike the Medtronic 3387/3389 DBS electrodes mate with their “Activa” series of implantable pulse generators. Such leads are currently being designed in our laboratory.

Next, a pulse generator must be constructed that is capable of producing and analyzing the FSCV waveform. Mayo Clinic has already designed and tested such a device (“WINCS”) along with a mating DBS stimulator (“MINCS”) (Chang et al., 2012a), and it is this WINCS device that was used to gather the data presented in this study. A second generation non-implantable but fully integrated FSCV/DBS device (sensing and stimulation) has also been constructed within our laboratory, and prototypes are currently undergoing testing at Mayo Clinic. A third generation of fully-implantable FSCV/DBS stimulators will also need to be developed before neurotransmitter-based feedback can be clinically useful. Design of this 3rd-generation implantable device will depend on data gathered with the non-implantable version, as implantability imposes significant constraints on size, reliability, and power consumption.

Finally, a control algorithm that can use FSCV-measured neurotransmitter concentration data to control DBS stimulation parameters in order to maintain a set neurotransmitter level must be designed. This algorithm is also under development within the Mayo Clinic Neural Engineering Lab. All of these elements must come together to enable a clinically-useful closed-loop DBS device based on neurotransmitter concentration sensing, but prior to this study there existed no obvious solution to the need for a durable, safe, and effective human FSCV electrode.

While CFMs provided excellent sensitivity for intraoperative use, their mechanical fragility complicated the surgery, and their electrochemical instability over time would never allow for the possibility of chronic implantation. By comparison, the properties of diamond-based electrodes largely overcome mechanical issues, while their electrochemical characteristics offer significant potential for chronic recordings *in vivo*. Furthermore, the correlation between observed tremor reduction and adenosine-like FSCV signature at the diamond electrode suggests that an FSCV-based closed-loop DBS system may well be within the reach of practical implementation.

## Materials and methods

### Chemicals

Chemicals were acquired from Sigma-Aldrich (St Louis, MO), and were used as received. All solutions were made using deionized (DI) water. Flow cell experiments were conducted in Tris buffer (150 mM sodium chloride and 12 mM Tris, pH adjusted to 7.4 with concentrated sodium hydroxide solution). For flow cell injection, 5 mM dopamine stock solution was prepared in DI water, and diluted to desired concentration in Tris buffer.

### Electrode fabrication

Both boron-doped diamond and CFMs were utilized in this study. To prepare boron-doped diamond microelectrodes, boron and carbon were co-deposited to form diamond films on tungsten wires (250 μm in diameter, A-M systems, Carlsborg, WA) using custom developed, hot-filament CVD techniques. The source gas was 1% methane in hydrogen with trimethylborane (1000 ppm in hydrogen) as the boron dopant source. The CVD reactor filament temperature was nominally maintained at 2000°C and the total pressure at 20 torr. Prior to CVD processing, the tungsten wires were electrochemically etched in 1 M sodium hydroxide by applying 10 V AC voltage to create a tapered sharpened tip. The generated tungsten tips were sonicated with 100 nm diamond particles suspended in isopropyl alcohol. To obtain the diamond growth temperature, the tungsten substrates were positioned 8–10 mm from the hot filament.

After CVD boron-doped diamond deposition, the coated tungsten wires were subjected to low pressure polymerization, resulting in a ~30 μm coating of parylene-C. This material provided enhanced biocompatibility as well as providing an insulating coating along the shaft of the electrode. Since the parylene uniformly coats the entire electrode during deposition, the first ~100 μm of the electrode tip's boron-doped diamond coating is subsequently exposed by selectively ablating the parylene coating using a pulsed ultraviolet laser (Photomachining, Inc. of Pelham, NH). The electrode was inserted into an outer cannula (FHC, Inc. Bowdoin, ME) that served as a stainless steel reference electrode.

For comparison, a CFM was fabricated by attaching a single polyacrylonitrile-based carbon fiber (7 μm in diameter; Cytec, Woodland Park, NJ) to a Nitinol (an alloy of nickel and titanium) extension wire with a silver-based conductive adhesive. The connection between the carbon fiber and the Nitinol wire was covered with cured polyamic acid (polyamide). The exposed carbon fiber was trimmed under a dissecting microscope to a length of 50–100 μm. The details of CFM production are discussed by Chang et al. (2012a).

### Electrode characterization

Surface morphology and microstructure of the boron-doped diamond electrode and CFM were imaged with a SEM (Hitachi S4700 Field Emission SEM) under conditions of 1.0 kV accelerating voltage and 10 μA beam current.

The confocal Raman measurements were acquired using an *alpha 300R WITec* system (Ulm, Germany) equipped with a UHTS300 spectrometer and a thermoelectrically cooled DV40-11 CCD detector. A frequency-doubled Nd:YAG laser at 532 nm was used for excitation. The samples were mounted on a piezoelectric, computer-controlled stage, with the film normal to the incident laser beam. To minimize the optical effects occurring from the inherent curvature of the tungsten rods, a Nikon 20x objective was employed.

The chemical morphology of diamond films was explored by 2-D surface confocal Raman mapping. To generate these Raman mapping images of 100 × 100 μm dimensions, a Raman spectrum was recorded at every image pixel, for a total of more than 100,000 spectra. Mappings of material constituents, namely diamond, boron, and sp^2^ type of carbon impurities, were first obtained using filters for their characteristic Raman vibrations at 1332 cm^−1^, centered around 550 and 1500 cm^−1^, respectively. The visual correlation of the spatial distribution of the above-mentioned constituents was accomplished by false coloring them with red (for diamond), blue (for boron), and green (for carbon impurities), and by merging these independent maps. To reduce the background noise, the intensity threshold for diamond, boron and non-diamond carbon peaks included in mapping was appropriately adjusted.

### Wireless instantaneous neurochemical concentration sensing (WINCS) system

FSCV detection of dopamine was achieved using WINCS. Briefly, WINCS hardware incorporates front-end analog transimpedance amplifier circuitry, a Bluetooth transceiver and a microcontroller—all integrated with a multilayer printed wiring board (PWB). The microcontroller produces an FSCV waveform applied to the electrochemical sensors, digitizes the nanoampere level electrochemical signal after current-voltage conversion by the transimpedance amplifier and controls the flow of data to the base station. Digital telemetry between the remote WINCS unit and base station is achieved by an embedded Bluetooth transceiver. WINCS software, “WincsWare,” (Mayo Clinic, Division of Engineering) controls the scan parameters and operation of WINCS, such as starting and stopping data acquisition and transmission, modifying FSCV waveform, changing sampling rate, and nearly real-time saving, conditioning, and displaying transmitted data.

### Flow injection analyses of dopamine

Both diamond and CFMs were calibrated with a custom designed flow cell. A FIAlab 3200 injection system (FIAlab Instruments, Seattle, WA) was utilized to introduce Tris buffer and dopamine sequentially to the sensing electrode. Dopamine samples with concentration ranging from 0.5 to 10 μM were used for calibration and were prepared by diluting 5 mM stock solution in Tris buffer. A triangular waveform was generated by WincsWare, with the potential ramped from −0.4 to +1.5 V and back at a scan rate of 400 V/s. This waveform was continually applied to the electrode at 10 Hz. Ag/AgCl served as the reference electrode. For each dopamine sample, five injections were attempted. The cyclic voltammograms prior to and after each injection were collected and subtracted from each other to obtain the cyclic voltammogram of dopamine. The oxidation peak currents for those injections were then averaged for calibration.

To evaluate their long term durability, both diamond microelectrode and CFMs were continually subjected to the triangular waveform described above at 10 Hz for 144 h. Dopamine calibrations were taken prior to 72 h and after 144 h of waveform application.

### *In vivo* neurotransmitter measurement

#### Consent process to recruit participants

The research protocol was approved by Mayo Clinic IRB (Institutional Review Board, protocol #09-007441). Outpatients in our neurosurgery clinic were given the opportunity to consent to the study before surgery. A mandatory waiting period of one night prior to their scheduled surgery was instituted to allow patients to withdraw if they elected to do so. The following was ensured:

1. Participants can knowledgeably discuss the consent document with their physician (assure understanding) and whomever else they turn to for advice (e.g., family members).
2. Participants understand that no services, treatments, or benefits will be withheld if they do not participate in the research. They also understand that their consent to the study will not directly benefit them.

#### Deep brain stimulation (DBS) surgery protocol inclusion/exclusion criteria

##### Inclusion

Adult patients with medically intractable essential tremor or Parkinson's disease who have been approved for DBS surgery by the interdisciplinary Mayo DBS committee.

##### Exclusion

Pregnant patients, prisoners, children (under 18 years of age), and any patients identified by the Mayo Clinic DBS committee as unsuitable for the study protocol.

#### DBS neurosurgery in human subjects

These studies were approved by Mayo Clinic IRB (IRB #09-007441) and registered with ClinicalTrials.gov (Clinical trials # NCT01705301). Written informed consent was obtained from all patients prior to surgery. Under local anesthesia, an MRI-compatible stereotactic head frame was fixed to the patient's head. A localizer box created nine fiducials as reference points to enable localization of MR images in stereotactic space. The patient was then transported to the MRI scanner. MR imaging was conducted using a General Electric Sigma 1.5 T MRI clinical system operated by EchoSpeed LX Version 9.1. The human DBS imaging protocol consists of MP-RAGE sequences using 1.5 mm slice thickness and 24 cm field of view. Using COMPASS navigational software, MRI data were merged with the human Schaltenbrand and Wahren stereotactic atlas, and stereotactic coordinates for DBS electrode implantation were identified. The patient was then returned to the operating suite where, under local anesthesia, a skin incision in line with the trajectory coordinates was made followed by a 5–10 mm burr hole made in the skull using a high-speed drill. Microelectrodes for standard electrophysiological recording and for the FSCV recording using a sensing probe, called a “WincsTrode,” were implanted simultaneously through 5-trajectory guide cannula system that was attached to the Alpha Omega microdrive system. As cellular activities were measured through the center trajectory to define the target, FSCV recordings were performed in a 2 mm anterior path from the center of the 5-trajectory guide cannula system. Once brain mapping was successfully performed, the electrophysiological recording electrode was replaced with the DBS electrode. Electrochemical recordings utilizing the WincsTrode were obtained to evaluate the concomitant changes in neurochemical extracellular levels.

To document potential microthalamotomy effects, the frequency and amplitude of hand tremor were recorded using a wireless accelerometer which was affixed to patients' wrists during surgery. To obtain a baseline, accelerometer recordings were made 20 s before DBS electrode implantation. During this DBS surgery in the operation room, FSCV recordings were performed using the WINCS system. There were no complications following DBS surgery and concurrent electrochemical recordings.

#### Fast scan cyclic voltammetry (FSCV) recording in humans

To perform FSCV recording, WINCS and WincsWare were utilized. For FSCV, the potential at the electrode was linearly scanned at 400 V/s in a triangular waveform from –0.4 to 1.5 V and back to –0.4 V at 10 Hz for co-monitoring, when present, adenosine and dopamine. The electrode rests at a bias potential of −0.4 V between scans.

#### Electrode and WINCS sterilization

Prior to implantation in patients, every effort was made to ensure safe and sterile implantation. WINCS units were sterilized by the Sterrad® hydrogen peroxide gas plasma process. Electrodes and accessory wires were sterilized with an ethylene oxide treatment. Ethylene oxide, the most common chemical sterilization method, is used for over 70% of all sterilizations and for 50% of all disposable medical devices. Ethylene oxide treatment was carried out for 24 h at 60°C with relative humidity above 30% and a gas concentration of 200 mg/l. This process was followed by a 72 h decay period in which the sterilized electrodes were quarantined. Because the pre- and post-sterilization calibrations were nearly identical, it appeared that the ethylene oxide sterilization did not affect the structure and characteristics of either the diamond or carbon fiber-based FSCV electrodes.

## Author contributions

KB contributed the initial concept of boron-doped diamond electrodes for human neurochemical detection, developed the diamond reactor, conceived project, critically revised manuscript, conceived Raman analysis procedure, and interpreted data. JT drafted and critically revised the manuscript, developed experimental design, and analyzed data. HM developed, analyzed and interpreted human FSCV data. FM developed the Raman analysis, interpreted the data, and drafted the Raman section of manuscript. MM fabricated electrodes, and analyzed longevity data. SP collected and analyzed animal and human studies data. MS operated the reactor, fabricated electrodes, and analyzed production data. EN collected and evaluated animal studies data. CB reviewed and critically revised the manuscript. AK provided critical revisions and interpretation of Raman data. SC developed, analyzed, and interpreted animal FSCV data. KL performed human surgeries, conceived of the application in humans, and drafted and revised the human section. The authors declare no competing financial interests.

### Conflict of interest statement

The authors declare that the research was conducted in the absence of any commercial or financial relationships that could be construed as a potential conflict of interest.

## References

[B1] Adams K. L. Puchades M. Ewing A. G. (2008). *In vitro* electrochemistry of biological systems. Annu. Rev. Anal. Chem. 1, 329. 10.1146/annurev.anchem.1.031207.113038 20151038 PMC2819529

[B2] Agnesi F. Tye S. J. Bledsoe J. M. Griessenauer C. J. Kimble C. J. Sieck G. C.. (2009). Wireless Instantaneous Neurotransmitter Concentration System-based amperometric detection of dopamine, adenosine, and glutamate for intraoperative neurochemical monitoring. J. Neurosurg. 111, 701–711. 10.3171/2009.3.JNS0990 19425899 PMC2814519

[B3] Bekar L. Libionka W. Tian G. F. Xu Q. Torres A. Wang X.. (2008). Adenosine is crucial for deep brain stimulation-mediated attenuation of tremor. Nat. Med. 14, 75–80. 10.1038/nm1693 18157140

[B4] Bernard M. Baron C. Deneuville A. (2004). About the origin of the low wave number structures of the Raman spectra of heavily boron doped diamond films. Diam. Relat. Mater. 13, 896–899. 10.1016/j.diamond.2003.11.082

[B5] Bitziou E. O'Hare D. Patel B. A. (2008). Simultaneous detection of pH changes and histamine release from oxyntic glands in isolated stomach. Anal. Chem. 80, 8733–8740. 10.1021/ac801413b 18947199

[B6] Bledsoe J. M. Kimble C. J. Covey D. P. Blaha C. D. Agnesi F. Mohseni P.. (2009). Development of the Wireless Instantaneous Neurotransmitter Concentration System for intraoperative neurochemical monitoring using fast-scan cyclic voltammetry. J. Neurosurg. 111, 712–723. 10.3171/2009.3.JNS081348 19425890 PMC2808191

[B7] Burns R. S. LeWitt P. A. Ebert M. H. Pakkenberg H. Kopin I. J. (1985). The clinical syndrome of striatal dopamine deficiency. Parkinsonism induced by 1-methyl-4-phenyl-1,2,3,6-tetrahydropyridine (MPTP). N. Engl. J. Med. 312, 1418–1421. 10.1056/NEJM198505303122203 2581135

[B8] Cahill P. S. Walker Q. D. Finnegan J. M. Mickelson G. E. Travis E. R. Wightman R. M. (1996). Microelectrodes for the measurement of catecholamines in biological systems. Anal. Chem. 68, 3180–3186. 10.1021/ac960347d 8797378

[B9] Chan H. Y. Aslam D. M. Wiler J. A. Casey B. (2009). A novel diamond microprobe for neuro-chemical and -electrical recording in neural prosthesis. J. Microelectromech. Syst. 18, 511–521. 10.1109/JMEMS.2009.2015493

[B10] Chang S. Y. Jay T. Munoz J. Kim I. Lee K. H. (2012a). Wireless fast-scan cyclic voltammetry measurement of histamine using WINCS–a proof-of-principle study. Analyst 137, 2158–2165. 10.1039/c2an16038b 22416270 PMC3360524

[B11] Chang S. Y. Kim I. Marsh M. P. Jang D. P. Hwang S. C. Van Gompel J. J.. (2012b). Wireless fast-scan cyclic voltammetry to monitor adenosine in patients with essential tremor during deep brain stimulation. Mayo Clin. Proc. 87, 760–765. 10.1016/j.mayocp.2012.05.006 22809886 PMC3538486

[B12] Cragg S. J. Hille C. J. Greenfield S. A. (2000). Dopamine release and uptake dynamics within nonhuman primate striatum *in vitro*. J. Neurosci. 20, 8209–8217. 11050144 10.1523/JNEUROSCI.20-21-08209.2000 PMC6772736

[B13] Dommett E. Coizet V. Blaha C. D. Martindale J. Lefebvre V. Walton N.. (2005). How visual stimuli activate dopaminergic neurons at short latency. Science 307, 1476–1479. 10.1126/science.1107026 15746431

[B14] Garris P. A. Kilpatrick M. Bunin M. A. Michael D. Walker Q. D. Wightman R. M. (1999). Dissociation of dopamine release in the nucleus accumbens from intracranial self-stimulation. Nature 398, 67–69. 10.1038/18019 10078530

[B15] Halpern J. M. Xie S. T. Sutton G. P. Higashikubo B. T. Chestek C. A. Lu H. (2006). Diamond electrodes for neurodynamic studies in Aplysia californica. Diam. Relat. Mater. 15, 183–187. 10.1016/j.diamond.2005.06.039

[B16] Hashemi P. Dankoski E. C. Petrovic J. Keithley R. B. Wightman R. M. (2009). Voltammetric detection of 5-hydroxytryptamine release in the rat brain. Anal. Chem. 81, 9462–9471. 10.1021/ac9018846 19827792 PMC2783829

[B17] Keithley R. B. Takmakov P. Bucher E. S. Belle A. M. Owesson-White C. A. Park J.. (2011). Higher sensitivity dopamine measurements with faster-scan cyclic voltammetry. Anal. Chem. 83, 3563–3571. 10.1021/ac200143v 21473572 PMC3089759

[B18] Lee K. H. Blaha C. D. Garris P. A. Mohseni P. Horne A. E. Bennet K. E.. (2009). Evolution of deep brain stimulation: human electrometer and smart devices supporting the next generation of therapy. Neuromodulation 12, 85–103. 10.1111/j.1525-1403.2009.00199.x 20657744 PMC2908254

[B19] Lee K. H. Blaha C. D. Harris B. T. Cooper S. Hitti F. L. Leiter J. C.. (2006). Dopamine efflux in the rat striatum evoked by electrical stimulation of the subthalamic nucleus: potential mechanism of action in Parkinson's disease. Eur. J. Neurosci. 23, 1005–1014. 10.1111/j.1460-9568.2006.04638.x 16519665

[B20] Lee K. H. Chang S. Y. Roberts D. W. Kim U. (2004). Neurotransmitter release from high-frequency stimulation of the subthalamic nucleus. J. Neurosurg. 101, 511–517. 10.3171/jns.2004.101.3.0511 15352610

[B21] Lee K. H. Kristic K. van Hoff R. Hitti F. L. Blaha C. Harris B.. (2007). High-frequency stimulation of the subthalamic nucleus increases glutamate in the subthalamic nucleus of rats as demonstrated by *in vivo* enzyme-linked glutamate sensor. Brain Res. 1162, 121–129. 10.1016/j.brainres.2007.06.021 17618941

[B22] Marcelli G. Patel B. A. (2010). Understanding changes in uptake and release of serotonin from gastrointestinal tissue using a novel electroanalytical approach. Analyst 135, 2340–2347. 10.1039/c0an00260g 20596571

[B23] McCreery D. B. Agnew W. F. Yuen T. G. Bullara L. A. (1995). Relationship between stimulus amplitude, stimulus frequency and neural damage during electrical stimulation of sciatic nerve of cat. Med. Biol. Eng. Comput. 33(3 Spec No), 426–429. 10.1007/BF02510526 7666690

[B24] Meng H. M. Wang Y. A. Huang M. Lin W. H. Wang S. Zhang B. M. (2011). Chronic deep brain stimulation of the lateral habenula nucleus in a rat model of depression. Brain Res. 1422, 32–38. 10.1016/j.brainres.2011.08.041 21978548

[B25] Navailles S. Benazzouz A. Bioulac B. Gross C. De Deurwaerdere P. (2010). High-frequency stimulation of the subthalamic nucleus and L-3,4-dihydroxyphenylalanine inhibit *in vivo* serotonin release in the prefrontal cortex and hippocampus in a rat model of Parkinson's disease. J. Neurosci. 30, 2356–2364. 10.1523/JNEUROSCI.5031-09.2010 20147561 PMC6634027

[B26] Park J. Show Y. Quaiserova V. Galligan J. J. Fink G. D. Swain G. M. (2005). Diamond microelectrodes for use in biological environments. J. Electroanal. Chem. (Lausanne. Switz.) 583, 56–68. 10.1016/j.jelechem.2005.04.032

[B27] Patel B. A. Bian X. H. Quaiserova-Mocko V. Galligan J. J. Swain G. M. (2007). *In vitro* continuous amperometric monitoring of 5-hydroxytryptamine release from enterochromaffin cells of the guinea pig ileum. Analyst 132, 41–47. 10.1039/B611920D 17180178

[B28] Phillips P. E. M. Stuber G. D. Heien M. L. A. V. Wightman R. M. Carelli R. M. (2003). Subsecond dopamine release promotes cocaine seeking. Nature 422, 614–618. 10.1038/nature01476 12687000

[B29] Rice M. E. Cragg S. J. Greenfield S. A. (1997). Characteristics of electrically evoked somatodendritic dopamine release in substantia nigra and ventral tegmental area *in vitro*. J. Neurophysiol. 77, 853–862. 9065854 10.1152/jn.1997.77.2.853

[B30] Roberts J. G. Moody B. P. McCarty G. S. Sombers L. A. (2010). Specific oxygen-containing functional groups on the carbon surface underlie an enhanced sensitivity to dopamine at electrochemically pretreated carbon fiber microelectrodes. Langmuir 26, 9116–9122. 10.1021/la9048924 20166750

[B31] Robinson D. L. Venton B. J. Heien M. L. Wightman R. M. (2003). Detecting subsecond dopamine release with fast-scan cyclic voltammetry *in vivo*. Clin. Chem. 49, 1763–1773. 10.1373/49.10.1763 14500617

[B32] Runnels P. L. Joseph J. D. Logman M. J. Wightman R. M. (1999). Effect of pH and surface functionalities on the cyclic voltammetric responses of carbon-fiber microelectrodes. Anal. Chem. 71, 2782–2789. 10.1021/ac981279t 10424168

[B33] Sanchez-Gonzalez M. A. Garcia-Cabezas M. A. Rico B. Cavada C. (2005). The primate thalamus is a key target for brain dopamine. J. Neurosci. 25, 6076–6083. 10.1523/JNEUROSCI.0968-05.2005 15987937 PMC6725054

[B34] Sarter M. Bruno J. P. Parikh V. (2007). Abnormal neurotransmitter release underlying behavioral and cognitive disorders: toward concepts of dynamic and function-specific dysregulation. Neuropsychopharmacology 32, 1452–1461. 10.1038/sj.npp.1301285 17164812

[B35] Shon Y. M. Chang S. Y. Tye S. J. Kimble C. J. Bennet K. E. Blaha C. D.. (2010a). Comonitoring of adenosine and dopamine using the Wireless Instantaneous Neurotransmitter Concentration System: proof of principle. J. Neurosurg. 112, 539–548. 10.3171/2009.7.JNS09787 19731995 PMC2852872

[B36] Shon Y. M. Lee K. H. Goerss S. J. Kim I. Y. Kimble C. Van Gompel J. J.. (2010b). High frequency stimulation of the subthalamic nucleus evokes striatal dopamine release in a large animal model of human DBS neurosurgery. Neurosci. Lett. 475, 136–140. 10.1016/j.neulet.2010.03.060 20347936 PMC2874873

[B37] Singh Y. S. Sawarynski L. E. Michael H. M. Ferrell R. E. Murphey-Corb M. A. Swain G. M.. (2010). Boron-doped diamond microelectrodes reveal reduced serotonin uptake rates in lymphocytes from adult rhesus monkeys carrying the short allele of the 5-HTTLPR. ACS Chem. Neurosci. 1, 49–64. 10.1021/cn900012y 20352073 PMC2843923

[B38] Staal R. G. Mosharov E. V. Sulzer D. (2004). Dopamine neurons release transmitter via a flickering fusion pore. Nat. Neurosci. 7, 341–346. 10.1038/nn1205 14990933

[B39] Suaud-Chagny M. F. (2004). *In vivo* monitoring of dopamine overflow in the central nervous system by amperometric techniques combined with carbon fibre electrodes. Methods 33, 322–329. 10.1016/j.ymeth.2004.01.009 15183181

[B40] Suzuki A. Ivandini T. A. Yoshimi K. Fujishima A. Oyama G. Nakazato T.. (2007). Fabrication, characterization, and application of boron-doped diamond microelectrodes for *in vivo* dopamine detection. Anal. Chem. 79, 8608–8615. 10.1021/ac071519h 17918970

[B41] Swamy B. E. K. Venton B. J. (2007). Subsecond detection of physiological adenosine concentrations using fast-scan cyclic voltammetry. Anal. Chem. 79, 744–750. 10.1021/ac061820i 17222045

[B42] Takmakov P. Zachek M. K. Keithley R. B. Walsh P. L. Donley C. McCarty G. S.. (2010). Carbon Microelectrodes with a Renewable Surface. Anal. Chem. 82, 2020–2028. 10.1021/ac902753x 20146453 PMC2838506

[B43] Wightman R. M. Robinson D. L. (2002). Transient changes in mesolimbic dopamine and their association with ‘reward’. J. Neurochem. 82, 721–735. 10.1046/j.1471-4159.2002.01005.x 12358778

[B44] Xie S. T. Shafer G. Wilson C. G. Martin H. B. (2006). *In vitro* adenosine detection with a diamond-based sensor. Diam. Relat. Mater. 15, 225–228. 10.1016/j.diamond.2005.08.018

[B45] Yoshimi K. Naya Y. Mitani N. Kato T. Inoue M. Natori S.. (2011). Phasic reward responses in the monkey striatum as detected by voltammetry with diamond microelectrodes. Neurosci. Res. 71, 49–62. 10.1016/j.neures.2011.05.013 21645558

[B46] Zhao H. Bian X. C. Galligan J. J. Swain G. M. (2010). Electrochemical measurements of serotonin (5-HT) release from the guinea pig mucosa using continuous amperometry with a boron-doped diamond microelectrode. Diam. Relat. Mater. 19, 182–185. 10.1016/j.diamond.2009.10.004 20209031 PMC2832314

