# Supplementary material for: A Diamond-Based Electrode for Detection of Neurochemicals in the Human Brain
Source: Front Hum Neurosci. 2016 Mar 15;10:102. doi: 10.3389/fnhum.2016.00102 (PMC4791376; doi:10.3389/fnhum.2016.00102)
Supplement: Supplementary file 1 [file DataSheet1.DOCX]

# Supplementary Materials

Four human volunteers were recruited for the initial trails of the diamond electrode from among patients undergoing deep brain stimulation electrode lead placement surgery at Mayo Clinic. These patients all were being treated for either Parkinson’s disease or essential tremor. While not all patients are addressed in the body of this paper, data from each of them is presented below. Figure S1 contains voltammograms from all 4 patients. Table T1 and Figure S2 describe the placement of the electrodes.


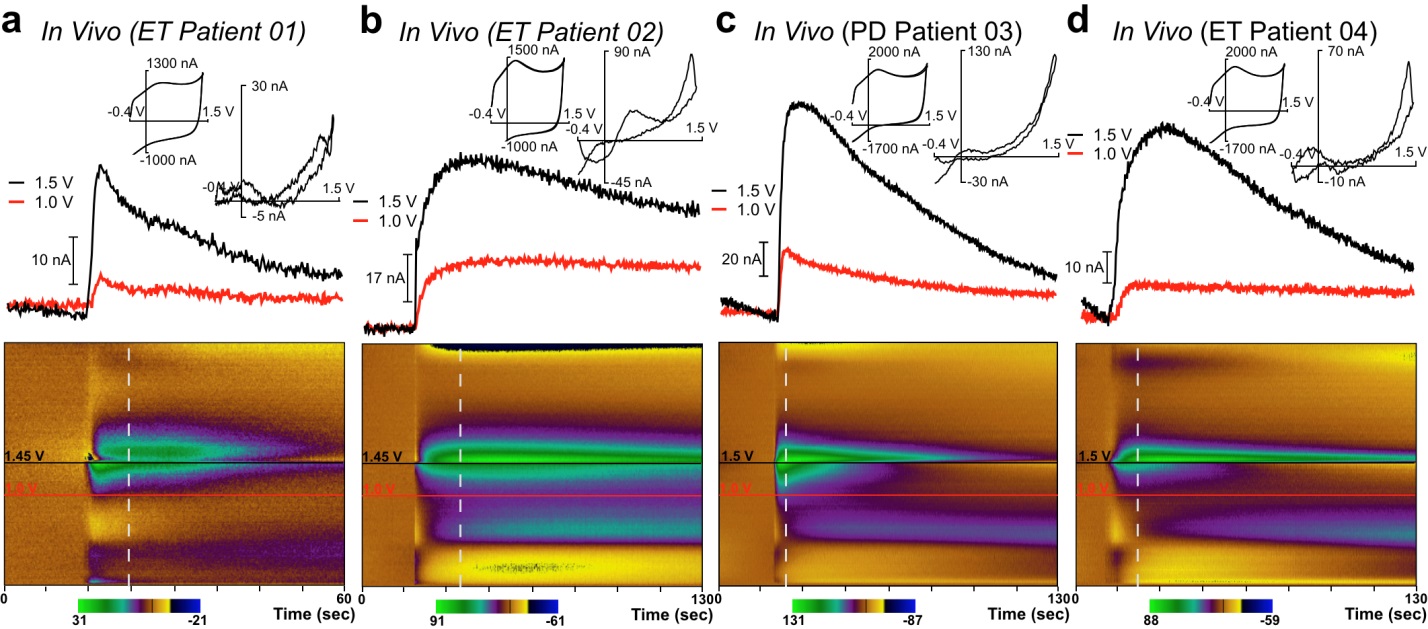


**Figure S1** – Data from all four human patients who received diamond electrodes are presented as panels (a) through (d). Patients 1, 2, and 4 were undergoing DBS lead implantation surgery for treatment of essential tremor, while patient 3 was being treated for Parkinson’s disease. For this reason, in patients 1,2, and 4 the electrode was placed in the ventral intermediate nucleus (VIM) of the thalamus while in patient 3 it was placed in the subthalamic nucleus – the sites of their (subsequent) DBS lead placement.

**Table T1** – Targeting information for each of the 4 patients.

| Patient Number | Subject Neurologic Condition | Recording Location | AC-PC Midline Coordinates (x, y, x) | Microelectrode Coordinates relative to AC-PC Midline (x, y, z) |
| --- | --- | --- | --- | --- |
| 1 | Essential Tremor | Ventral Intermediate Nucleus of the Thalamus | 0, 0, 0 | 14.3, -7.97, 2.1 |
| 2 | Essential Tremor | Ventral Intermediate Nucleus of the Thalamus | 0, 0, 0 | 18.75, -7.93, -3.16 |
| 3 | Parkinson’s Disease | Subthalamic Nucleus | 0, 0, 0 | 14.5, -2.47, 4.09 |
| 4 | Essential Tremor | Ventral Intermediate Nucleus of the Thalamus | 0, 0, 0 | 11.04, -7.31, 2.84 |


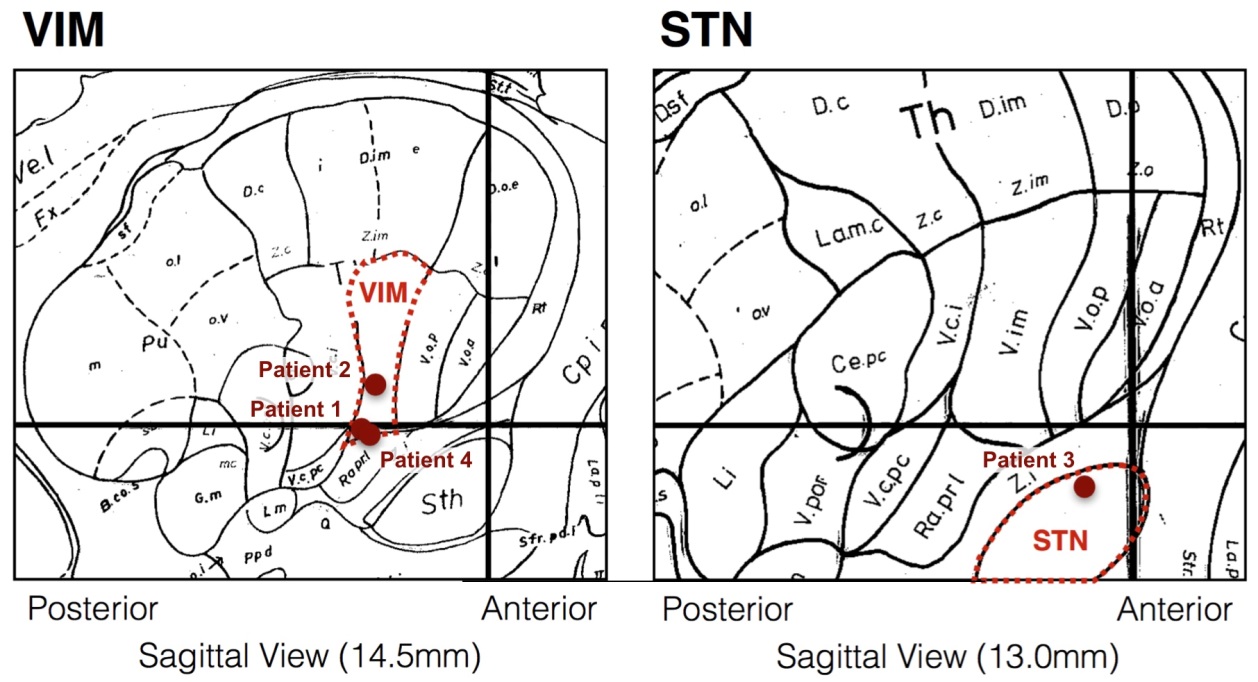


**Figure S2** – Targeting information (superimposed on a brain atlas) for each of the 4 patients. The Parkinson’s disease patient corresponds to the subthalamic nucleus panel, while the ventral intermediate nucleus of the thalamus was the target in the three essential tremor patients.
